# Demographic and Psychological Predictors of Panel Attrition: Evidence from the New Zealand Attitudes and Values Study

**DOI:** 10.1371/journal.pone.0121950

**Published:** 2015-03-20

**Authors:** Nicole Satherley, Petar Milojev, Lara M. Greaves, Yanshu Huang, Danny Osborne, Joseph Bulbulia, Chris G. Sibley

**Affiliations:** 1 The University of Auckland, Auckland, New Zealand; 2 Victoria University of Wellington, Wellington, New Zealand; University of Westminster, UNITED KINGDOM

## Abstract

This study examines attrition rates over the first four years of the New Zealand Attitudes and Values Study, a longitudinal national panel sample of New Zealand adults. We report the base rate and covariates for the following four distinct classes of respondents: explicit withdrawals, lost respondents, intermittent respondents and constant respondents. A multinomial logistic regression examined an extensive range of demographic and socio-psychological covariates (among them the Big-Six personality traits) associated with membership in these classes (*N* = 5,814). Results indicated that men, Māori and Asian peoples were less likely to be constant respondents. Conscientiousness and Honesty-Humility were also positively associated with membership in the constant respondent class. Notably, the effect sizes for the socio-psychological covariates of panel attrition tended to match or exceed those of standard demographic covariates. This investigation broadens the focus of research on panel attrition beyond demographics by including a comprehensive set of socio-psychological covariates. Our findings show that core psychological covariates convey important information about panel attrition, and are practically important to the management of longitudinal panel samples like the New Zealand Attitudes and Values Study.

## Introduction

Studies based on longitudinal self-report questionnaires are vital for understanding numerous processes in psychology and related fields. Such studies allow for the examination of change in individuals over time, the stability of measures, and causal inference [1]. Nevertheless, the strength of longitudinal panel designs rests or falls on their ability to retain participants over time. The main concern here is that the loss of participants (i.e., sample attrition) can be systematic rather than random. For example, older people are more likely to own their own homes relative to younger people, who are more prone to change their address. As a result, it may be more difficult to contact and retain younger (vs. older) people in longitudinal research. This would result in selective attrition, whereby younger individuals are lost from the study at a higher rate than older individuals. In other words, longitudinal samples may over-represent—or under-represent—certain groups of individuals over time. This may introduce systematic bias to relevant estimates and threaten the validity of findings generated. It is therefore critical to understand the factors associated with selective attrition so that such issues can be addressed in longitudinal research.

Beyond the immediate implications for a given longitudinal study, selective attrition provides insight into research participation as a general behaviour. That is, we can gain a comprehensive understanding of the underlying characteristics of distinct types of respondents. In turn, this ability to differentiate between different types of respondents allows us to provide stronger inferences about the reasons why people participate in longitudinal research. This allows numerous questions to be raised. For example, how do personality traits influence participants’ inclination to respond? Are altruistic individuals more likely to respond given their pro-social attitudes? Are patriotic individuals more likely to consistently respond than their less patriotic counterparts? Answering such questions may help guide retention strategies used in longitudinal studies. Unfortunately, this opportunity has been largely overlooked in past research.

Here, we document the base rates of different forms of attrition, as well as their demographic and psychological covariates, in the New Zealand Attitudes and Values Study (NZAVS). The NZAVS is a longitudinal national panel survey of New Zealand adults who were originally sampled via postal mail from the New Zealand Electoral Roll (i.e., a national registry of voters). The aim of the NZAVS is to understand how and why the health, wellbeing, personality, attitudes and values of New Zealanders change over time. The NZAVS was started in 2009, is conducted in yearly waves, and is currently into its sixth year of a planned 20 in total. In terms of personality research alone, longitudinal data from the NZAVS has provided a foundation to assess short-form scale reliability over time [2], the curvilinear nature of personality stability across age cohorts [3], as well as the stability of Big-Six personality traits in the face of disruptive environmental events (i.e., the 2011 Christchurch earthquake) [4]. Although univariate differences in key measures between those who did and did not complete the waves necessary for such analyses have been noted on a case-by-case basis (e.g., [4]), a comprehensive examination of selective attrition in the NZAVS has remained lacking.

In the current study, we aim to determine whether particular measures in the NZAVS are associated with panel members’ propensity to respond across the first four years of the study’s projected 20 year life-span. Accordingly, we define four classes of respondents: constant respondents, intermittent respondents, explicit withdrawals, and lost respondents (see Table 1 for descriptions). The former two classes represent forms of retention, whereby commitment to the study has been expressed beyond the first wave. The latter two classes constitute forms of attrition. After documenting overall retention rates and the frequency of these different types of respondents, we examine an extensive range of covariates (e.g., demographic, socio-psychological and personality factors) that may predict differential class membership.

**Table 1:** Formal definitions for different response classes.

Attrition	
Name	Definition
Explicit Withdrawals	Actively withdrew from the study at any time following Time 1.
Lost Respondents	Has not responded following Time 1, but not explicitly withdrawn

Retention	
Name	Definition
Intermittent Respondents	Responded to any 2 or 3 waves, including Time 1
Constant Respondents	Responded at every wave from Time 1—Time 4

Notes: These response classes refer only to response patterns over the first 4 waves (Time 1—Time 4) of the NZAVS. The Explicit Withdrawals response class excludes withdrawals due to death.

Assessing selective attrition in this way highlights the demographic and socio-psychological characteristics unique to each response class. That is, our approach allows us to estimate which variables are associated with a higher or lower likelihood of attrition overall, as well as the specific form of attrition. This, in turn, provides an indication of which measures might produce biased estimates in longitudinal research, as well as indicating the extent of the potential problem. As such, we contribute to the extant literature by consolidating findings on the demographic predictors of sample attrition, while also providing a comprehensive set of socio-psychological predictors of attrition.

### Methods in Previous Research on Attrition in Panel Surveys

Internationally, a considerable amount of research has investigated selective attrition in longitudinal studies across a diverse range of survey formats. Many of these studies include various combinations of face-to-face and telephone interviews, as well as written and online survey responses. Such methods have been seen in the likes of internet panels (namely the Longitudinal Internet Studies for the Social Sciences or LISS, [5], [6]), birth cohort studies [7] and household studies [8], to name a few. Moreover, studies often differ in the type of attrition they analyse. Rather than pooling all forms of attrition into an overall measure, some studies have examined separate avenues of attrition. These include mortality, inability to make contact, and general refusal to continue with, or active withdrawal from the study [9], [10]. Consequently, standards of reporting sample attrition have varied to a large extent in past literature.

Despite this variation, the analytic methods used to predict panel member loss have (by and large) been very similar. Given the strong focus on differentiating those who remain in the study from those who cease their participation, comparisons of those who respond to a follow-up wave and those who do not have been prevalent (e.g., [8], [11]). However, a small number of studies have diverged from this common approach of dichotomizing a participant’s response state by employing continuous measures of research participation. For example, Porter and Whitcomb [12] used the number of surveys a participant completed as a measure of their underlying level of cooperation, whereas others have examined panel duration [6] and survival processes [6], [7].

Another method used by fewer still involves outlining a respondent typology. This method tends to better-reflect the multi-wave design of longitudinal data, whereby participants may respond to some, but not all, waves of a study. In using this approach, Ware et al. [13] found that, relative to constant respondents, the magnitude of risk ratios for a variety of measures was lesser for intermittent respondents than for those who missed all follow-up waves. In a more recent and comprehensive examination of respondent typology, Lugtig [5] employed Latent Class Analysis to identify the attributes of 9 distinct classes of respondents who differed in their probabilities of responding to a given wave of the LISS.

Such research has been insightful in identifying intermittent respondents as a distinct respondent group. Intermittent responding may be problematic for longitudinal analyses because it means fewer waves of data are available for some participants, resulting in less power to detect effects. Beyond concerns about statistical power, identifying the correlates of intermittent responding should provide an additional aid for understanding the factors that influence a participant’s inclination to participate in—and commit to—a panel study. This is because intermittent responding reflects a unique group of respondents, who neither participate in full, nor completely exit, the study. With this in mind, the present study employs a similar typology-based approach by specifying intermittent respondents as a distinct response class. Consequently, we contribute to the relatively limited area of research addressing this type of response pattern.

### Demographics and Attrition

Socio-demographic factors are by far the most studied predictors of panel attrition. Despite the diversity in survey formats used, findings on the influence of these measures have been relatively consistent. For example, international research indicates that attrition tends to be higher among non-whites relative to whites [11], [13], [14], men relative to women [7], [11], and singles relative to those in relationships [11], as well as the less educated [5], [7], [8], [10]. In terms of age, both younger and older people often drop out of longitudinal studies [14], possibly due to contact rates and health issues (respectively). Another largely consistent finding is that there is a lower response rate among those living in urban, as opposed to rural, areas [6], [14].

Although the general trends in panel attrition across nations are well documented, the size (and even direction) of a specific predictor’s effect on attrition can vary across countries [15]. Consequently, it is important to assess selective attrition in a wide range of national and cultural settings. In this respect, New Zealand presents a unique social context to examine sample attrition. The ethnic group composition of the country, which consists of three main minority groups (i.e., Māori, Asian, and Pacific peoples) alongside the majority NZ European group, is one aspect of this context. Māori are the indigenous peoples of New Zealand and tend to be over-represented in deprived regions of the country [16]. On average, Māori tend to experience poorer health and greater susceptibility to psychological distress relative to NZ Europeans [17]. While different ethnic groups have differing social circumstances, past research on attrition with regards to ethnicity has (for the most part) focused exclusively on the distinction between Whites and non-Whites, which are oftentimes broadly-defined minorities (e.g., [11]). Such wide-stroked categorizations may overlook important differences between minority groups.

Initial insight into the New Zealand context may be found in cross-sectional survey responses. Looking at the NZAVS 2009 base sample, approximately 81.9% of respondents reported NZ European as their ethnicity, 17.9% reported being Māori, 4.3% Pacific, and 4.9% Asian. By comparison, of the New Zealand Census indicates that NZ Europeans, Māori, Pacific, and Asians constitute approximately 75.1%, 11.9%, 5.7%, and 11.7% (respectively) of the New Zealand population who are aged 18 and older [18]. In terms of gender, 40.5% of NZAVS respondents were men (vs. 59.5% women) compared to 47.9% (vs. 52.1% women) in the adult NZ population [18].

This reveals a tendency for women, NZ Europeans, and Māori (albeit due to targeted sampling frames) to be overrepresented in the NZAVS base sample. Men, Pacific Islanders, and Asian peoples in particular are underrepresented. This is despite an additional sampling frame targeting the above minority ethnic groups. Similar patterns can be seen in other cross-sectional surveys such as the New Zealand Election Study (NZES), which conducts postal surveys following each general election in New Zealand. The NZES reported 46.6% of their respondents were men (versus 51.7% women) in their 2011 survey [19]. Percentages of ethnic group identification show that 79.2% of respondents reported their ethnic group identification as NZ European, 11.4% as Māori, 4.8% as Asian, and 2.6% as Pacific (NZES, 2011). Thus, the 2011 NZES contains sampling discrepancies that are similar to the NZAVS. Such sampling biases may be compounded in longitudinal studies once one takes into account the cumulative effects of non-random attrition.

### Beyond Demographic Predictors of Attrition

Research into additional predictors of panel attrition has focused on a wide range of factors, albeit in less depth. Health is a predictor of attrition, such that participants who report better subjective health are more likely to respond to follow-up waves [11]. In contrast, those who smoke are less likely to remain in a study [10]. Other research has focused on participants’ subjective experience with the survey. These studies demonstrate that participants who have a more enjoyable experience as reported both directly and indirectly (in having a more experienced interviewer) are more likely to respond to follow-up waves [20], [21]. Further, Loosveldt, Pickery, and Billiet [22] showed that item non-response on an initial survey is predictive of later refusal to a follow-up survey, a finding that has been widely supported by others (e.g., [13], [8]).

By comparison, socio-psychological predictors have received surprisingly little attention in the literature, perhaps due to a lack of longitudinal studies that focus on relevant constructs. However, research that has examined socio-psychological correlates of attrition show that low levels of social support are associated with attrition [10], as is being highly self-interested [23]. These findings seem to better-demonstrate the social processes involved in people’s decision to participate in a panel study, reflecting the role of social capital (or a lack of), as well as the perceived benefits of participation. Indeed, social processes are central to many theories on panel management. Estrada, Woodcock, and Wesley Schultz [24], for example, have developed Tailored Panel Management (TPM) as an approach to longitudinal research design that emphasizes the ‘communal exchange’ between researcher and participant. They suggest that the panel represents a social community and that participation is an act of reciprocity built on established norms (that is, responding at the researcher’s request). Others suggest that principles of reciprocity are only effective in motivating participation for those who feel a sense of belonging in society [6]. Examining the socio-psychological predictors of attrition within a comprehensive framework should advance such knowledge and theories on panel participation.

### Personality Factors and Sample Attrition

The Big-Five personality traits (Openness, Conscientiousness, Extraversion, Agreeableness, and Emotional Stability; [25], [26]) are reliable markers of personality that have been used to explain a broad range of behaviours and phenomena within many disciplines. More recently, the HEXACO model of personality (i.e., the Big-Six), which incorporates Honesty—Humility as an additional sixth trait, updates the Big-Five to provide a better account of findings about the factor structure of personality derived from lexical studies [27]. Given the utility of personality traits as a broad predictor of behaviour, relatively little attention has been paid to how they might explain research participation. Attrition selective on personality traits would further demonstrate the universal applicability of traits for understanding social phenomena. Indeed, in a related study, Dollinger and Leong [28] found Agreeableness, Openness to Experience, and Extraversion to be positively associated with volunteering for follow-up longitudinal research. However, these findings may reflect people’s motivation to seek out new experiences, rather than maintaining one’s current commitment to participating in an ongoing longitudinal study.

Such long-term commitment should be linked to the trait of Conscientiousness. Conscientiousness tends to be associated with diligence, orderliness, and a motivation to carry out and complete tasks [27] which, in the case of a longitudinal study, would involve completing surveys. Supporting this notion, Lugtig [5] found that the Big-Five could distinguish respondent classes. Specifically, he found that those in the class with the highest response probabilities (‘loyal stayers’) were higher on Conscientiousness than both those who maintained lower response probabilities (‘lurkers’, who were higher on Extraversion) and those who stopped responding (‘fast attriters’, who were higher on Agreeableness). On the other hand, Saßenroth [6] failed to find a link between Conscientiousness and panel duration in the LISS. Extraversion and Openness to Experience were, however, associated with panel attrition in the study [6].

Finally, Richter, Körtner, and Saßenroth [29] analysed response rates in the German Socio-Economic Panel study. They found that Openness had the most reliable, albeit small effect on panel duration across different subsamples of respondents. They also found that Extraversion and Conscientiousness were positively associated with panel duration amongst the older subsample (i.e., those who had been panel members the longest), although these effects were attenuated when adjusting for age and sex. Taken together, these findings demonstrate a relative lack of consensus among the scarce research on personality and sample attrition.

Critically, no research to date has examined panel attrition within the framework of the HEXACO model of personality. This is a notable absence, given that Honesty—Humility is characterized by sincerity and unassuming willingness to cooperate [27]. Accordingly, those who are higher on Honesty-Humility should be more willing to help out with and contribute to the survey process, and thus respond more frequently. Considering their sincerity, they may also feel less hesitant to provide genuine responses, which may lead to a greater affinity towards responding. By assessing the effects of the Big-Six on attrition, our investigation provides further insights into the effects of personality on attrition beyond those offered in extant research.

### Overview and Guiding Hypotheses

In the current study, we examine predictors of response class in the NZAVS. Specifically, we seek to identify whether an extensive range of demographic, as well as socio-psychological, measures are predictive of participants’ response class (constant respondents, intermittent respondents, explicit withdrawals, lost respondents) during the first four waves of the study. In doing so, we model the log-odds of class membership relative to a baseline class (the constant respondent class) as a function of our covariates. As such, our obtained odds ratios provide the odds of being in each respondent class, relative to the constant respondent class, for each of our predictor variables (e.g., men relative to women).

We include numerous socio-psychological measures potentially relevant to individuals’ inclination to maintain participation in a longitudinal study. Given the gaps in the extant literature discussed above, we include the Big-Six personality markers in our analyses. We expect constant respondents to be higher on Conscientiousness than intermittent and lost respondents. To a lesser degree, Extraversion and Honesty—Humility should also be associated with response class, whereby classes marked by attrition should be positively associated with Extraversion, and negatively with Honesty—Humility.

Adopting the view of a panel as a social community, we also include measures pertaining to the social aspects of panel participation. People who have a low sense of belonging may view the panel as a social group or community, and therefore be more inclined to participate to bolster their sense of belonging. Similarly, people with a low sense of support may see participation in the panel as a source of social support. We therefore assess the potential effects of sense of belonging and sense of support.

Past research (as discussed earlier) has linked self-interest with attrition [23], which we further explore by including both self-enhancement and altruistic values. Surveys can be time-consuming to complete and often offer little incentive in return, which people who are driven by self-enhancement motives may be particularly sensitive to. As such, these individuals may be more likely to cease participation. Conversely, the pro-social nature of individuals with altruistic values may make them more willing to volunteer their time to complete surveys that will benefit the researcher and hopefully the wider community. Life satisfaction could influence an individual’s interest in committing to a panel study. For example, people with low levels of life satisfaction may be more concerned with fulfilling basic needs than completing a survey. This could lead to a negative association between life satisfaction and the constant respondent class. Finally, because patriotism is associated with greater civic participation and characterised by an attachment to one’s country [30], we might expect the breadth of the NZAVS and its interests specific to New Zealand to appeal to those higher in patriotism.

Aside from socio-psychological covariates we also coded whether people provided an email address, cell phone number, and landline phone number in addition to their postal address. Each of these contact methods presents a unique way of maintaining contact with participants. These avenues of contact should be linked to greater participant retention, and therefore positively associated with the constant respondent class. Finally, we were particularly interested in the links between sample attrition and demographic factors unique to the social context of New Zealand. Specifically, we predicted that Māori, Pacific Islanders, and Asian peoples would be more likely than NZ Europeans to belong to the intermittent respondent and the attrition classes, relative to the constant respondent class.

## Method

### Sample and Participants

The Time 1 (2009) NZAVS contained responses from 6,518 participants sampled from the 2009 New Zealand electoral roll. Of these, complete data for the covariates included in our analysis was available for 5,814 people (see Table 2 for demographic details). We limited our analysis to these 5,814 people. The electoral roll is publicly available for scientific research and, in 2009, contained 2,986,546 registered voters. This represented all citizens over 18 years of age who were eligible to vote (regardless of whether they chose to vote), barring those who had their contact details removed due to specific case-by-case concerns about privacy. The sample frame was spilt into three parts. Sample Frame 1 constituted a random sample of 25,000 people from the electoral roll (4,060 respondents). Sample Frame 2 constituted a second random sample of an additional 10,000 people from the electoral roll (1,609 respondents). Sample Frame 3 constituted a booster sample of 5,500 people who were randomly selected from meshblock area units of the country with a high proportion of Māori, Pacific Nations and Asian peoples (671 respondents). A further 178 people responded but did not provide contact details and so could not be matched to a sample frame. In sum, postal questionnaires were sent to 40,500 registered voters (or roughly 1.36% of all registered voters in New Zealand). The overall response rate (adjusting for the address accuracy of the electoral roll and including anonymous responses) was 16.6%.

**Table 2:** Summary sample details for the first four yearly waves of the NZAVS (2009–2012).

	Time 1	Time 2	Time 3	Time 4
Sample size during wave	6518	4442	6884	12182
Additions during wave	-----	19	2965	5378
Retained from wave 1 (Time 1, %)	-----	67.86	60.06	62.18
Retained from previous wave (%)	-----	67.86	79.47	83.70
Retained from at least one previous wave (%)	-----	67.86	59.94	68.56
Demographics (Percentages)				
New Zealand Europeans	81.9	85.9	74.8	84.4
Māori	17.9	15.5	10.8	16.6
Pacific Nations peoples	4.3	3.6	2.6	5.0
Asian peoples	4.9	4.0	3.7	5.1
Gender (Women)	59.5	61.6	62.5	62.5
Age (Mean)	48	51	52	49

Note: Additions to a particular wave include booster sampling, opt-ins, and un-matched participants.

**Fig 1:**
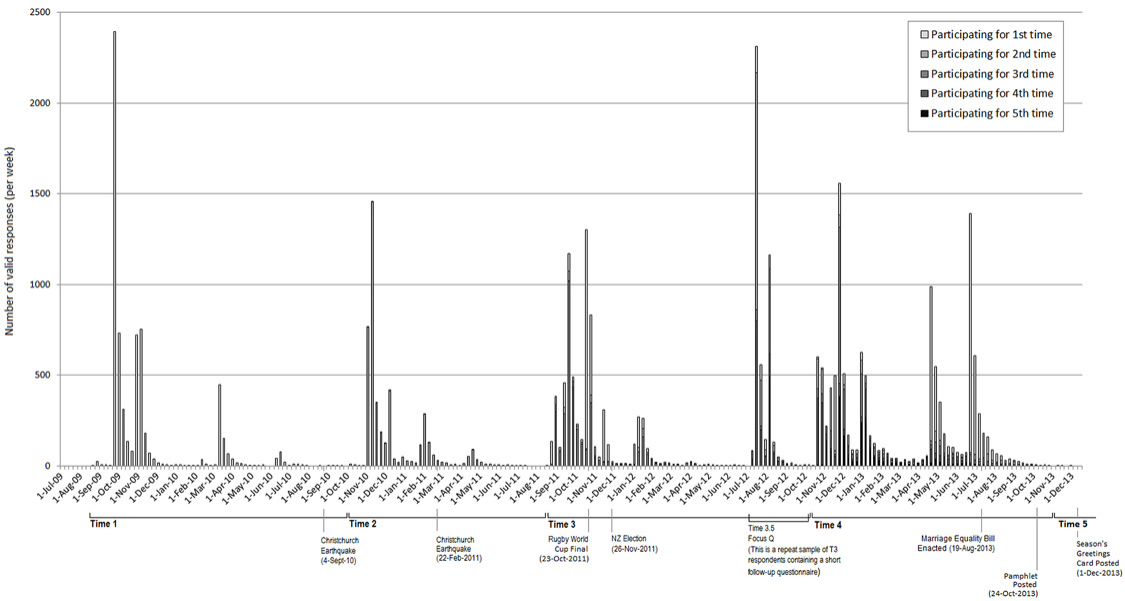
Response timeline for Time 1—Time 4 of the NZAVS.

While our analyses only utilise data from those sampled at Time 1 of the NZAVS, data collection has been completed up to Time 4. Fig. 1 displays a timeline of NZAVS data collection, including the number of responses received per week during each wave of data collection, as well as the number of times participants had responded. It also lists some of the larger events occurring in New Zealand during the lifetime of the NZAVS thus far. This highlights a somewhat hidden utility of longitudinal designs, whereby data collection is happening before, during, and after various societal events that may shape the way people are responding. As such, questionnaire responses can be examined for change in relation to these events.

### NZAVS Sampling and Retention Strategy

Following the second wave of data collection, the NZAVS introduced a number of strategies aimed to help improve retention rates. These strategies were introduced following the second wave of the study due to a relaxation in initial funding constraints and are briefly described below.

For each wave of the NZAVS, participants received a paper questionnaire each year via postal mail. From Time 3 onwards, participants also received a pre-notification email and were also provided a link to an online version of the questionnaire. This email stated that a paper copy of the questionnaire would be on its way to them through the post shortly, but that if they preferred, they could also complete the questionnaire online using the link provided. If a response to the postal or online questionnaire was not received within three months of initial contact, then participants received another duplicate questionnaire and email (if they had provided an email address). Starting at Time 4, participants who failed to respond to this subsequent questionnaire were phoned.

Phoning participants has since been a key retention strategy for the NZAVS. Most participants provided a contact number (either a landline and or mobile phone number) on their questionnaire consent form. Non-respondents were phoned up to three times using each available phone number. If informed that a participant was deceased, he or she was marked in a database as deceased. If a participant withdrew during the phone call, a research assistant requested a reason for withdrawal. Subsequently, types of withdrawal were coded as either ‘self-selected out’ or ‘withdrawn due to illness.’ Participants who expressed a reluctance to participate were promptly told their details would be removed for one year, and received a questionnaire at the next wave, so as to not over burden them.

It should be noted that many participants responded positively to the phone call and either updated their address details or requested a new copy of the questionnaire. If this was the case, a new questionnaire was then either posted or emailed to them. If participants could not be contacted by phone (either they had not provided phone numbers, their phone number had changed, or they were unavailable), then they were sent a follow-up questionnaire and/or email near the end of the yearly wave.

The NZAVS has adopted several other retention strategies over the first few waves of the study as more funding has become available. One strategy that started in 2013 (i.e., Time 4) was to send a pamphlet regarding the current progress of the study to all participants via post. The 2013 pamphlet featured a cover-photo of many of the key research team, and inside it displayed simple graphs and accessible summaries of prominent NZAVS research, alongside a brief letter from the principal investigator thanking participants. From 2013 onwards a Season’s Greetings card was also mailed out, which asked participants to update their contact details to make sure they would be eligible for one of five prize draws for $200 of grocery vouchers. The season’s greeting card was designed specifically for the NZAVS. An archive of these materials by Huang, Greaves, and Sibley [31] can be found on the NZAVS website.

### Questionnaire Measures

Big-Six personality traits were assessed using the Mini-IPIP scales presented in Donnellan et al. [32] and adapted by Sibley et al. [33] to form the Mini-IPIP6 with the addition of items that assessed Honesty-Humility. Respondents were asked to “Please circle the number that best represents how accurately each statement describes you” on a scale from 1 (very inaccurate) to 7 (very accurate). The items for each Big-Six personality trait and their associated scale Cronbach’s alpha are as follows: Extraversion: “Am the life of the party”; “Don't talk a lot” (reverse coded); “Keep in the background” (reverse coded); “Talk to a lot of different people at parties” (α = .71). Agreeableness: “Sympathize with others' feelings”; “Am not interested in other people's problems” (reverse coded); “Feel others' emotions”; “Am not really interested in others” (reverse coded, α = .66). Conscientiousness: “Get chores done right away”; “Like order”; “Make a mess of things” (reverse coded); “Often forget to put things back in their proper place” (reverse coded, α = .65). Neuroticism: “Have frequent mood swings”; “Am relaxed most of the time” (reverse coded); “Get upset easily”; “Seldom feel blue” (reverse coded, α = .64). Openness to Experience: “Have a vivid imagination”; “Have difficulty understanding abstract ideas”; “Do not have a good imagination”; “Am not interested in abstract ideas” (where the latter three were reverse coded, α = .67). Honesty-Humility: “Feel entitled to more of everything”; “Deserve more things in life”; “Would like to be seen driving around in a very expensive car”; “Would get a lot of pleasure from owning expensive luxury goods” (all reverse coded, α = .78).

The scales for sense of belonging and support were adapted from Cutrona and Russell [34], and Williams, Cheung, and Choi [35]. To measure sense of belonging, participants were asked to “Please circle the number that best represents how accurately each statement describes you” on a scale from 1 (very inaccurate) to 7 (very accurate) for the following items: “Know that people in my life accept and value me”; “Feel like an outsider” (reverse coded); “Know that people around me share my attitudes and beliefs” (α = .53). To assess sense of support participants were asked to “Please indicate how strongly you agree or disagree with each statement” on a scale from 1 (strongly disagree) to 7 (strongly agree) for the following items: “There are people I can depend on to help me if I really need it”; “There is no one I can turn to for guidance in times of stress” (reverse coded); “I know there are people I can turn to when I need help” (α = .75).

Life satisfaction was assessed using two items from the scale developed by Diener, Emmons, Larsen, and Griffin [36]. Participants were asked to “Please indicate how strongly you agree or disagree with each statement” on a scale from 1 (strongly disagree) to 7 (strongly agree) for the following two items: “I am satisfied with my life” and “In most ways my life is close to ideal” (α = .76).

Patriotism was measured using two items from Kosterman and Feshbach [30]. Participants were asked to “Please indicate how strongly you agree or disagree with each statement” on a scale from 1 (strongly disagree) to 7 (strongly agree): “Although at times I may not agree with the government, my commitment to New Zealand always remains strong” and “I feel a great pride in the land that is our New Zealand” (α = .69).

Altruistic and self enhancement values were assessed using marker items from Schwartz’s [37] scales. For each set of scale items, participants were asked to “Please circle the number that best represents how important each of the following values is for you as a guiding principle in your life” for the following items: Altruistic values: “EQUALITY (equal opportunity for all)”; “A WORLD AT PEACE (free of war and conflict)”; “SOCIAL JUSTICE (correcting injustice, care for the weak)” (α = .71), and Self-enhancement values: “AUTHORITY (the right to lead or command)”; “INFLUENCE (having an impact on people and events)”; “WEALTH (material possessions, money)” (α = .61).

### Neighbourhood-level deprivation

We measured the affluence of participants’ immediate (small area) neighborhood using the New Zealand Deprivation Index [38]. New Zealand is unusual in having rich census information about each area unit/neighborhood of the country available for research purposes. The smallest of these area units are meshblocks. Statistics New Zealand [39] describes a meshblock as “a defined geographic area, varying in size from part of a city block to large areas of rural land. Each meshblock abuts against another to form a network covering all of New Zealand including coasts and inlets, and extending out to the two hundred mile economic zone”. The geographical size of these meshblock units differs depending on population density, but each unit tends to cover a region containing a median of roughly 90 residents (M = 103, SD = 72, range = 3–1,431).

The 2006 New Zealand Deprivation Index [38] uses aggregate census information about the residents of each meshblock to assign a decile-rank score ranging from 1 (most affluent) to 10 (most impoverished) to each meshblock unit. Because it is a decile-ranked index, the 10% of meshblocks that are most affluent are given a score of 1, the next 10% a score of 2, and so on. The index is based on a principal components analysis of the following nine variables (in weighted order): (a) proportion of adults who received a means-tested benefit, (b) household income, (c) proportion who do not own their own home, (d) proportion who are single-parent families, (e) proportion who are unemployed, (f) proportion who are lacking qualifications (i.e., low educational status), (g) proportion who live in crowded household conditions, (h) proportion with no telephone access, and (i) proportion with no car access. The New Zealand Deprivation Index thus reflects the average level of deprivation for small neighborhood-type units (or small community areas) across the entire country.

The index is a well-validated measure of the level of deprivation of small area units, and has been widely used in health and social policy research examining numerous health outcomes, including mortality, rates of hospitalization, smoking, cot death, and access to health care, to name just a few examples [40–42]. The index is also widely used in service planning by government and local councils, and is a key indicator used to identify high needs areas and allocate resources such as health funding (see [43], [16]). The current sample had a mean deprivation index of 5.06 (SD = 2.85).

### Ethics statement

The data reported in this study were collected as part of a larger research project, the New Zealand Attitudes and Values Study. The study was approved by The University of Auckland Human Participants Ethics Committee on 09-September-2009, reference number: 2009/336. The study was re-approved by the University of Auckland Human Participants Ethics Committee on 17-February-2012 until 09-September-2015. Reference number: 6171. All participants gave written consent. Participants provided consent when completing the questionnaire, in their own time, and in their own space. The University of Auckland Human Participants Ethics Committee approved this consent procedure.

## Results

### Descriptive Statistics

**Fig 2:**
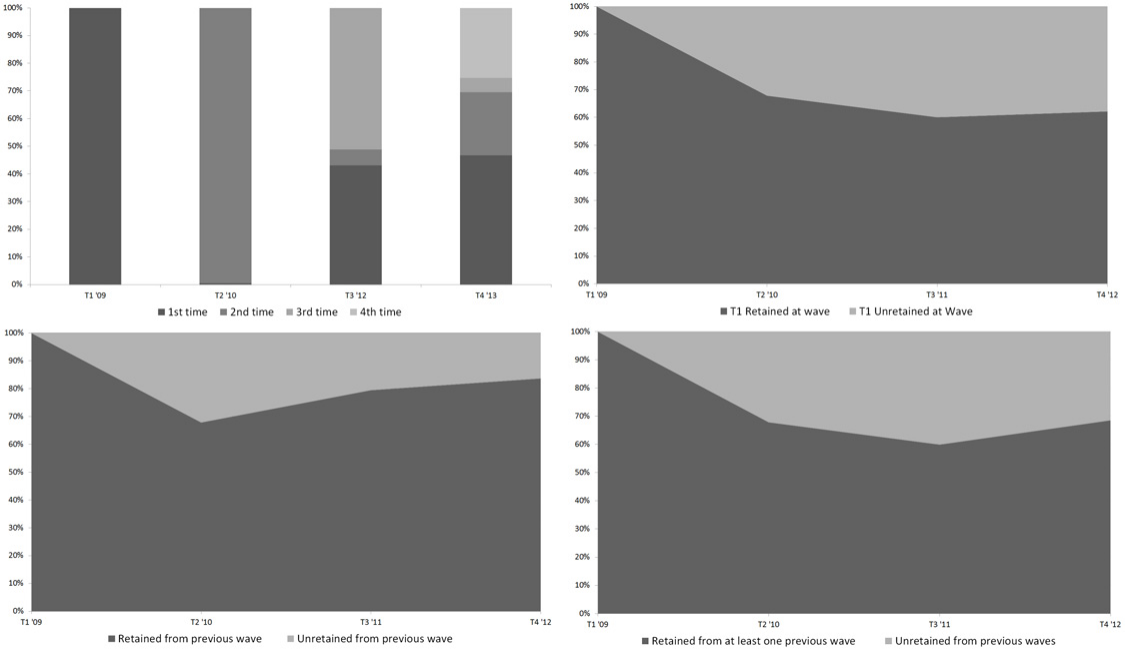
Graphs depicting the percentage breakdown of times responded by participants at each wave (upper left), percentage of Time 1 respondents retained vs. lost at each wave (upper right), the percentage of respondents retained vs. lost from the previous wave at the subsequent wave (lower left), and the percentage of respondents to any prior wave retained vs. lost at the given wave (lower right).

To present a broader view of sample retention and attrition in the NZAVS, details about the sample at each wave are displayed in Table 2, including key demographic measures at each wave. Table 2 also includes various measures of retention, such as the percentage of Time 1 respondents retained at each wave, wave-to-wave retention, and an overall measure of retention of those who participated in any previous wave, up to the given wave. This information is also depicted graphically in Fig. 2. The top left-hand graph depicts the percentage of respondents at each wave (Time 1—Time 4) who were participating for their first to fourth time. The upper right-hand graph displays retention of Time 1 respondents at each wave. The lower left-hand graph displays the percentage of respondents at each wave who were retained from the previous wave, relative to the percentage from the previous wave who did not complete the subsequent wave. Finally, the lower right-hand graph displays the percentage of respondents at each wave who were retained from previous waves, relative to the percentage of respondents at any previous wave who did not complete the given wave.

As shown in Fig. 2, retention rates generally improved at Time 4. Previous wave sample retention increased from 79.47% at Time 3 to 83.7% at Time 4, whereas retention of the original sample of participants at Time 1 (62.18%) increased relative to the percentage retained at Time 3 (60.06%). Moreover, a greater percentage of respondents were retained (68.56%) from any of the previous waves at Time 4 relative to any other time point. The improvement in retention likely reflects improvements made in retention strategies that occurred specifically during data collection at Time 4, when phoning was first introduced.

Focusing on the sample at hand, the 6,385 respondents to Time 1 of the NZAVS consisted of 499 explicit withdrawals, 1,065 lost participants, 1,733 intermittent respondents, and 3,088 constant respondents. This excludes the 133 participants who were listed as deceased at any point between Time 2 and Time 4 in order to focus on types of attrition that can be corrected or adjusted for by panel management. Percentages of ethnic groups and gender, as well as the mean age for each respondent class, are displayed in Table 3. Equality of these measures across columns would suggest there is no relationship between the given variable and respondent class. However, discrepancies are evident. Specifically, a greater percentage of constant respondents are NZ European (88.7%), whereas a larger percentage of lost and intermittent respondents identify as Māori (25.6% and 22.6% respectively), compared to the 13.1% of constant respondents who identify as Māori. Moreover, a higher percentage of constant respondents are women (62.7%) relative to the remaining response classes. Further, lost and intermittent respondents are notably younger (M_ages_ = 40.2 and 44.2, respectively) than those who belong to the remaining response classes.

**Table 3:** Percentages of demographic variables for each response class.

	Explicit Withdrawals	Lost Respondents	Intermittent Respondents	Constant Respondents
NZ European	79.2	70.8	78	88.7
Māori	13	25.6	22.6	13.1
Pacific	3.4	7.7	5.2	2.8
Asian	7.6	8.0	6.1	2.9
Other	5	3.8	2.8	3.5
Gender (female)	58.1	56.0	57.9	62.7
Age (mean)	53.1	40.2	44.2	51.1

Note: N(Explicit withdrawals) = 499.

N(Lost Participants) = 1065, N(Intermittent Respondents) = 1733.

N(Constant Respondents) = 3088.

Respondents could report more than one ethnicity.

### Logistic Regression Predicting Panel Attrition

To assess the association between our measures and response class, a multinomial logistic regression was conducted with response class as the dependent variable. A sample size of 5,814 was available for the analysis (we used listwise deletion of participants with missing data on the predictors in our model). The sample analysed here thus consisted of 447 explicit withdrawals, 884 lost respondents, 1,595 intermittent respondents, and 2,888 constant respondents. Constant respondents were used as the reference category for the analysis. As such, we modelled the log-odds of belonging to each of the remaining response classes (intermittent respondents, lost respondents, and explicit withdrawals) relative to the constant respondent class. This provides the change in log-odds associated with a one-unit increase in a given measure, with all else held constant. The results of this regression are reported in Table 4. Given the number of effects reported, we limit our discussion of the model to our central findings.

**Table 4:** Multinomial logistic regression model predicting response class, where the constant respondent class is the reference category.

	Explicit Withdrawals				Lost Respondents				Intermittent Respondents			
	b	se	z	OR [95% CI]	b	se	z	OR [95% CI]	b	se	z	OR [95% CI]
Threshold	-.307	.746			2.297	.584			1.062	.475		
Gender (Women 0, Men 1)	-.067	.119	-.560	.936 [.741, 1.181]	.312	.095	3.277**	1.366 [1.133, 1.646]	.193	.074	2.594**	1.213 [1.048, 1.403]
Employment (Employed 0, Unemployed 1)	.102	.131	1.330	1.108 [.857, 1.431]	-.027	.111	-.243	.973 [.782, 1.211]	.057	.086	.660	1.059 [0.894, 1.254]
Parental Status (Parent 0, Not a parent 1)	.179	.134	1.330	1.195 [.919, 1.555]	-.357	.113	-3.158**	.700 [.560, .873]	-.197	.092	-2.142*	.821 [.686, .983]
Relationship Status (Partner 0, No partner 1)	-.006	.128	-.049	.994 [.773, 1.278]	.003	.104	.026	1.003 [.818, 1.229]	.042	.083	.510	1.043 [.887, 1.227]
Country of Birth (Born in NZ 0, Not born in NZ 1)	.116	.139	.834	1.123 [.855, 1.474]	.352	.115	3.063**	1.422 [1.135, 1.781]	.148	.089	1.657	1.159 [.973, 1.381]
Religious (Religious 0, Not Religious 1)	-.053	.110	-.486	.948 [.765 [1.176]	-.097	.089	-1.087	.907 [.761, 1.081]	-.086	.069	-1.250	.918 [.802, 1.050]
Residence (Urban 0, Rural 1)	-.059	.109	-.542	.943 [.761, 1.167]	-.211	.091	-2.303*	.810 [.677, .969]	-.045	.069	-.658	.956 [.835, 1.094]
Māori (NZ European 0, Māori 1)	-.094	.164	-.573	.910 [.660 [1.256]	.465	.111	4.173**	1.592 [1.280, 1.981]	.423	.090	4.691**	1.526 [1.279, 1.821]
Asian (NZ European 0, Asian 1)	.793	.246	3.220**	2.210 [1.364, 3.581]	.489	.208	2.358*	1.631 [1.086, 2.450]	.413	.169	2.442*	1.511 [1.085, 2.106]
Pacific (NZ European 0, Pacific 1)	-.296	.303	-.977	.744 [.411, 1.347]	.071	.188	.378	1.074 [.742, 1.553]	-.044	.178	-.249	.957 [.674, 1.357]
Other (NZ European 0, Other 1)	.412	.251	1.639	1.509 [.923, 2.469]	-.044	.247	-.179	.957 [.589, 1.554]	-.441	.211	-2.092*	.643 [.426, .972]
Age	-.003	.005	-.682	.997 [.987, 1.006]	-.062	.004	-15.208**	.940 [.932, .947]	-.032	.003	-10.888**	.968 [.962, .974]
Education	-.229	.047	-4.868**	.795 [.725, .872]	-.203	.037	-5.471**	.816 [.759, .878]	-.064	.028	-2.265*	.938 [.887, .991]
Deprivation Score	-.012	.020	-.617	.988 [.950, 1.027]	.045	.016	2.807**	1.046 [1.014, 1.079]	.017	.012	1.328	1.017 [.992, 1.042]
Percentage of missing likert item responses	.076	.016	4.738**	1.079 [1.046, 1.114]	.061	.017	3.611**	1.063 [1.028, 1.098]	.018	.015	1.160	1.018 [.988, 1.049]
Email at T1 (0 No, 1 Yes)	-.431	.130	-3.319**	.650 [.504, .838]	-.319	.109	-2.917**	.727 [.587, .901]	-.285	.089	-3.185**	.752 [.631, .896]
Cell phone at T1 (0 No, 1 Yes)	-.146	.121	-1.207	.864 [.681, 1.096]	-.222	.103	-2.162*	.801 [.655, .989]	.126	.081	1.561	1.134 [.968, 1.328]
Landline at T1 (0 No, 1 Yes)	.014	.190	.076	1.014 [.700, 1.471]	-.678	.113	-6.005**	.508 [.407, .634]	-.271	.102	-2.655**	.762 [.624, .931]
Extraversion	-.077	.048	-1.624	.925 [.843, 1.016]	.134	.041	3.296**	1.144 [1.056, 1.239]	.028	.032	.876	1.028 [.966, 1.094]
Agreeableness	-.110	.062	-1.770	.896 [.793, 1.012]	-.016	.050	-.311	.985 [.893, 1.086]	-.034	.039	-.875	.967 [.896, 1.043]
Conscientiousness	-.071	.052	-1.370	.931 [.841, 1.031]	-.194	.041	-4.773**	.824 [.761, .892]	-.133	.032	-4.176**	.875 [.822, .932]
Neuroticism	.054	.055	.979	1.055 [.948, 1.175]	.097	.044	2.226*	1.102 [1.012, 1.200]	.023	.034	.679	1.023 [.957, 1.094]
Openness	-.050	.051	-.980	.951 [.861, 1.051]	-.033	.042	-.798	.967 [.891, 1.050]	.069	.032	2.139*	1.072 [1.006, 1.142]
Honesty—Humility	-.095	.043	-2.201*	.909 [.835, .990]	-.094	.036	-2.597**	.910 [.848, .977]	-.053	.029	-1.838	.948 [.896, 1.004]
Altruism Values	-.060	.050	-1.181	.942 [.853, 1.040]	.027	.039	.694	1.027 [.952, 1.109]	-.001	.030	-.018	.999 [.942, 1.060]
Self-Enhancement Values	.147	.044	3.336**	1.158 [1.063, 1.263]	.122	.037	3.294**	1.130 [1.051, 1.215]	.128	.028	4.550**	1.137 [1.076, 1.201]
Sense of Belonging	.079	.065	1.207	1.082 [.952, 1.230]	.205	.051	4.034**	1.228 [1.111, 1.356]	.172	.040	4.275**	1.187 [1.097, 1.284]
Sense of Support	-.067	.052	-1.284	.936 [.845, 1.036]	-.084	.043	-1.970*	.920 [.846, 1.000]	-.076	.034	-2.226*	.927 [.867, .991]
Life Satisfaction	.034	.056	.610	1.035 [.928, 1.154]	-.114	.043	-2.687**	.892 [.820, .970]	-.055	.034	-1.604	.946 [.884, 1.012]
Patriotism	.023	.059	.388	1.023 [.911, 1.150]	.002	.045	.049	1.002 [.918, 1.094]	.006	.036	.162	1.006 [.938, 1.079]

Note:

*p <. 05.

** p <.01.

N(Constant respondents) = 2888.

N(Explicit Withdrawals) = 447.

N(Lost participants) = 884.

N(Intermittent respondents) = 1595.

Model estimated using Maximum Likelihood with robust estimation of standard errors.

AIC = 12748.15.

BIC = 13368.27.

loglikelihood = -6281.07.

### Demographic Factors

Numerous demographic variables were reliably associated with respondent class membership. For example, significant gender differences were evident, such that men were 1.4 times as likely as women to be lost respondents (b = .312, se = .095, Z = 3.28, OR = 1.37, p = .001), and 1.2 times as likely to be intermittent respondents (b = .193, se = .074, Z = 2.59, OR = 1.21, p = .009). However, no significant difference between men and women was found for the explicit withdrawal response class (b = -.067, se = .119, Z = -.560, OR = .936, p = .575).

A number of ethnic group differences were also identified. In particular, the odds associated with Māori being lost respondents were 1.6 times those of NZ Europeans (b = .465, se = .111, Z = 4.17, OR = 1.59, p <. 001), and 1.5 times the odds of NZ Europeans being an intermittent respondent (b = .423, se = .090, Z = 4.69, OR = 1.53, p <. 001). In contrast, no significant differences between Māori and NZ Europeans were found in terms of belonging to the explicit withdrawal class (b = -.094, se = .164, Z = -.573, OR = .910, p = .567). Additionally, relative to NZ Europeans, Asian peoples were 2.2 times as likely to be in the explicit withdrawal class (b = .793, se = .246, Z = 3.22, OR = 2.21, p = .001), 1.6 times as likely to be in the lost respondent class (b = .489, se = .208, Z = 2.36, OR = 1.63, p = .018), and 1.5 times as likely to be in the intermittent respondent class (b = .413, se = .169, Z = 2.44, OR = 1.51, p = .015). While those whose ethnicity was classified as “other” were less likely than NZ Europeans to be intermittent respondents (b = -.441, se = .211, Z = -2.09, OR = .643, p = .036), no remaining significant differences in class membership were found between NZ Europeans and Pacific peoples, nor those whose ethnicity was classified as other (all p’s >.05). Together with gender, ethnic group differences tended to be the most pronounced of the demographic variables.

Contactability (email, landline, and cell-phone provision) was also associated with response class. Providing an email address at Time 1 was associated with significantly lower odds of belonging to the explicit withdrawal class (b = -.431, se = .130, Z = -3.32, OR = .650, p = .001), lost respondent class (b = -.391, se = .109, Z = -2.92, OR = .727, p = .004), and intermittent respondent class (b = -.285, se = .089, Z = -3.19, OR = .752, p = .001). Providing a landline phone Time 1 was also associated with significantly lower odds of being a lost respondent (b = -.678, se = .113, Z = -6.01, p <. 001), and intermittent respondent (b = -.271, se = .102, Z = -2.66, p = .008), while providing a cell phone number at Time 1 was only associated with significantly lower odds of belonging to the lost respondent class (b = -.222, se = .103, Z = -2.16, p = .031).

Overall, selective attrition was most evident in the lost respondent class. Beyond the effects described above, parents, those not born in NZ, the younger, the less educated, and those living in more deprived areas were more likely to be lost than constant respondents (all p’s <. 01). The effects of these demographic variables were generally weaker and less widespread for the explicit withdrawal class. Beyond the association with Asian peoples, education was the only other measure significantly associated with explicit withdrawals at the p <. 01 level, whereby greater education was associated with lower odds of being in the explicit withdrawal class relative to the constant respondent class (b = -.229, se = .047, Z = -4.87, OR = .795, p <. 001). Across all demographic measures, employment status, relationship status, and religious identification were the only measures unassociated with response class.

### Socio-psychological Measures

Numerous effects were also found for socio-psychological variables. Among the Big-Six personality traits, Conscientiousness and Honesty–Humility stood out as the most influential. Each one-unit increase in Conscientiousness was associated with a. 194 decrease in the log-odds of being a lost (vs. constant) respondent (b = -.194, se = .041, Z = -4.77, OR = .824, p <. 001), and a. 133 decrease in the log-odds of being an intermittent respondent (b = -.133, se = .032, Z = -4.18, OR = .875, p <. 001). Conscientiousness was, however, unassociated with the odds of being in the explicit withdrawal class (relative to the constant respondent class). On the other hand, Honesty-Humility was significantly associated with the explicit withdrawal and lost respondent classes. Each one-unit increase in Honesty-Humility was associated with a. 095 decrease in the log-odds of being an explicit withdrawal (b = -.095, se = .043, Z = -2.20, OR = .909, p = .028), and a. 094 decrease in the log-odds of being a lost respondent (b = -.094, se = .036, Z = -2.6, OR = .910, p = .009).

Increases in both Extraversion (b = .134, se = .041, Z = 3.30, OR = 1.14, p = .001) and Neuroticism (b = .097, se = .044, Z = 2.23, OR = 1.10, p = .026) were associated with greater log-odds of being a lost respondent, whereas an increase in Openness was associated with greater log-odds of being an intermittent respondent (b = .069, se = .032, Z = 2.14, OR = 1.07, p = .032). Agreeableness, however, was unassociated with membership in a given response class.

Beyond the Big-Six personality traits, self-enhancement values were significantly associated with each of the explicit withdrawal, lost respondent, and intermittent respondent classes. For each one-unit increase in self-enhancement values, the log-odds of explicitly withdrawing increased by. 147 (b = .147, se = .044, Z = 3.34, OR = 1.16, p = .001), and the log-odds of being a lost respondent increased by. 122 (b = .104, se = .037, Z = 3.29, OR = 1.13, p = .001). Likewise, the log-odds of being an intermittent respondent increased by. 128 (b = .128, se = .028, Z = 4.55, OR = 1.14, p <. 001) for every one-unit increase in self-enhancement values.

Sense of belonging was also a significant predictor of being a lost and intermittent (vs. constant) respondent, such that each one-unit increase in sense of belonging was associated with a. 205 increase in the log-odds of being a lost respondent (b = .205, se = .051, Z = 4.03, OR = 1.23, p <. 001), and a. 172 increase in the log-odds of being an intermittent respondent (b = .172, se = .040 Z = 4.28, OR = 1.19, p <. 001). Conversely, each one-unit increase in sense of support was associated with a. 084 decrease in the log-odds of being a lost respondent (b = -.084, se = .043, Z = -1.97, OR = .920, p = .049), and a. 076 decrease in the log-odds of being an intermittent respondent (b = -.076, se = .034 Z = -2.23, OR = .927, p = .026). Life satisfaction, however, was only significantly negatively associated with the lost respondent class relative to the constant respondent class (b = -.114, se = .043 Z = -2.69, OR = .892, p = .007). Neither altruistic values, nor patriotism, were significantly associated with belonging to any response class (all p’s >. 05).

## Discussion

We examined attrition rates over the first four years of the NZAVS. We also determined which demographic and socio-psychological variables were associated with attrition through their association with four response classes. In particular, we aimed to build on the emerging literature on the association between personality and sample attrition by examining the effects of the Big-Six personality traits. Findings are discussed in the following sections with reference to extant research.

### General Retention Rates

Retention improved at Time 4 of the NZAVS relative to earlier waves. However, rates of retention within the NZAVS still tended to be lower than rates reported in large-scale panel studies conducted in other countries. For example, Schoeni, Stafford, McGonagle, and Andreski [21] compiled data on a number of national panel studies including the GSOEP, HRS, HILDA, NLSY79, and PSID studies, and found that wave-to-wave retention rates were high; 88% at worst and 99% at best. By comparison, wave-to-wave retention at Time 4 of the NZAVS was 83.7%. However, this was an increase of approximately 4 and 16 percentage points compared to wave-to-wave retention at Time 3 and Time 2, respectively. Although increases in wave-to-wave response rates can be expected due to selective attrition [21], in the case of the NZAVS, these increases in retention likely occurred due to improvements in panel management. For example, the phoning procedures utilised for non-respondents, as detailed earlier, were introduced during data collection at Time 4 of the study. This was made possible due to an increase in resources behind the study and likely contributed the most to this improvement in retention.

In terms of the retention of the sample from the initial wave of the study, the NZAVS also saw a minor increase in retention such that 62.2% of Time 1 respondents responded to Time 4 (up from 60.1% at the prior wave). Again, this figure tends to be lower than those seen in other panel studies. For example, retention of the first wave respondents at the fourth wave of the HILDA panel was 76% [8], and 82.9% for the PSID study in the United States [44]. However, neither of those studies saw an increase in retention in subsequent waves, again suggesting that improvements in panel management in particular led to the improvements in retention seen in the NZAVS.

### Demographic Factors Linked to Panel Attrition

Our results indicated that, overall, ethnicity, gender, education, and age were most strongly associated with response class such that ethnic minorities, men, the less educated, and younger people were generally the least likely to be constant respondents. Demographic variables (but also socio-psychological variables) were most widely associated with the lost vs. constant respondent comparison, followed by the intermittent respondent class comparison. Encouragingly, the explicit withdrawal class was the least distinct from the constant respondent class across measures, which consisted of those respondents who permanently withdrew from the panel.

With regards to specific effects, our findings are in many ways consistent with past research on panel attrition. For example, lost respondents were more likely than constant respondents to be men, parents, those not born in New Zealand, those living in urban areas, ethnic minorities (i.e., Māori and Asian peoples), as well as the younger, less educated, and those living in more deprived areas. These associations suggest that, as is often assumed, participants in less stable living arrangements are most at risk of being lost or dropping out of longitudinal research. While most demographic factors were associated with at least one response class comparison, no signs of attrition were found for religious identification, employment status, or relationship status, suggesting that these measures should be free of attrition bias in related analyses.

Our results also show that the provision of contact details is positively associated with constant responding. More specifically, the provision of an email address was positively associated with constant responding (while adjusting for the presence or absence of a landline phone). Furthermore, having a cell phone number was negatively associated with belonging to the lost respondent class. Not surprisingly, this suggests that attrition caused by participants changing addresses can be buffered through the collection of their cell phone number.

By examining multiple distinct ethnic groups, we also identified specific differences in how minority groups are likely to respond to panel studies. In particular, relative to NZ Europeans, both Māori and Asian peoples (but not Pacific peoples) were more likely to be lost and intermittent respondents. Curiously, Asian peoples were the only ethnic group more likely than NZ Europeans to explicitly withdraw from the study. The reason behind this remains unclear. Given the nature of the NZAVS (a highly self-reflective survey for New Zealanders), this effect could be related to differences in perceptions of national identity, or cultural differences in the value of individual-focused, introspective exercises. In any case, our results highlight the importance of examining distinct ethnic groups separately, as differing patterns of attrition can emerge.

### Personality and Socio-Psychological Factors Linked to Panel Attrition

In examining the Big-Six personality traits, our results suggest that Honesty-Humility is associated with panel attrition. Specifically, constant respondents were higher on Honesty-Humility than both explicit withdrawals and lost respondents. Constant respondents were also higher on Conscientiousness than intermittent and lost respondents. This closely resembles the findings of Lugtig [5] whose loyal stayers (those with high response probabilities and who are conceptually similar to our constant respondents) were more conscientious than both lurkers (similar to our intermittent respondents, and who were also higher on Extraversion, much like our intermittent respondents) and participants who ceased responding (reflecting our lost respondent class). The high alignment between our response class definitions and the classes utilised by Lugtig likely contributed to these similarities. Unlike Lugtig, however, we failed to find evidence of an effect of Agreeableness on respondent class membership. Further, our findings differed in key respects from those of Richter, Körtner, and Saßenroth [29], who found that Openness was the strongest personality trait predictor of attrition across different subsamples of respondents. Indeed, we found little evidence of an association between Openness and panel attrition. This is perhaps unsurprising, given that their analysis differed considerably from the one presented here.

We also identified numerous socio-psychological predictors of respondent class membership. Together with Conscientiousness, self-enhancement values and sense of belonging were the strongest socio-psychological predictors of response class. The effect size for these variables was often as large as those seen for many of the demographic variables, if not larger—bearing in mind they were measured on a continuous scale, rather than the dichotomous scale used to assess most demographic variables. The positive association between self-enhancement values and attrition mimics findings in previous studies (e.g., [23]). While it also seemed plausible that altruistic individuals would be more willing to offer their time to complete surveys, we found no evidence of altruistic values being linked to response class. Intermittent and lost respondents tended to be higher on sense of belonging, although they also tended to be lower on sense of support, relative to constant respondents.

Estrada et al. [24] suggested that panels can be seen as a form of social community. As such, those with a low sense of belonging may recognise this and participate to bolster their sense of belongingness. However, our findings for sense of support contradict this notion. One possible explanation, given the subjectivity of the measure, is that a low sense of support actually stems from (at least in part) a lack of trust and willingness to confide in others, as opposed to the degree of actual support that is available. These feelings could generalise to concerns about disclosing responses to surveys, leading to the greater likelihood of attrition. Again, there appears to be no obvious explanation for such findings, and these suggestions are highly speculative.

### Recommendations and Implications

Selective attrition is important to recognise in longitudinal research as it may introduce systematic error to relevant parameter estimates. Indeed, many studies have found bias in estimates for measures associated with attrition (e.g., [5]). Further, Gray et al. [14] have shown that conclusions from analyses involving predictors of attrition can change when comparing results between initial respondents and retained respondents at a later wave. Conversely, Fitzgerald, Gottschalk, and Moffitt [44] have shown (perhaps encouragingly) that regressions on measures influenced by selective attrition reveal a greater impact of attrition on the intercept, whereas slope values remain unchanged (in most cases). This would suggest relationships between measures tend to remain the same. Though the effects of selective attrition on estimates are not straightforward or uniform across analyses, it is nevertheless important to determine where selective attrition is occurring and maintain awareness of these potential problems.

With this in mind, our analyses provide an important resource for longitudinal research, particularly in New Zealand where documentation of selective attrition has been lacking. This is especially true for studies in the early stages, such as the Growing Up in New Zealand Study [45], which could possibly benefit from knowledge of predictors of sample attrition. With regards to ethnicity, it seems that Māori and Asian peoples in particular are susceptible to attrition in longitudinal studies, with Asian peoples being more likely to completely cease their participation. As such, while targeted sampling frames may boost the representation of minority ethnic groups in an initial sample, our results suggest that this representation will be lost over time. Accordingly, care should be taken to ensure these groups have strong representation in initial waves of longitudinal studies.

Although our analyses did not test whether selective attrition resulted in biased estimates, our findings highlight the importance of considering a range of different measures when examining attrition in longitudinal studies. Given that many socio-psychological effects held when statistically adjusting for an extensive range of demographic variables, considering only demographics may not be enough to ensure adequate representation in a panel across all measures. Indeed, because population estimates for socio-psychological measures are not obtainable, such selective attrition may prove particularly problematic (i.e., it is difficult to conceive of how one could employ sample weights to correct for sampling biases in personality). However, examining selective attrition on socio-psychological measures in particular is beneficial in consolidating and aiding in the creation of strategies that promote retention in panel designs. For example, our results underscore the role of incentives in panel participation, owing to the effect of self-enhancement values, whereas altruistic individuals are seemingly no more likely to participate on goodwill alone.

### Limitations

A potential caveat to our analyses is the low initial response rate of 16.6% at Time 1 of the NZAVS. This may be due in part to the length of the NZAVS questionnaire, as well as the fact that participants were being requested to provide their details and agree to be contacted for the projected 20 year duration of the study. This low initial response rate may lead to questions surrounding whether our sample is typical of the target population, and therefore demonstrates typical or atypical patterns and predictors of attrition which could be expected from the target population. As noted earlier, some discrepancies exist between Time 1 of the NZAVS and census data in terms of ethnicity and gender. However, given that the effects obtained in our analyses are largely as hypothesised, it seems unlikely that such discrepancies influenced the results in any obvious or drastic way. Indeed, it may be that some effect sizes are actually underestimated. For example, it is quite possible that our sample is more conscientious than the general population, meaning the effect sizes for Conscientiousness predicting response class in the general population could be higher than estimated in our analysis.

It is also worth noting that analyses of attrition are conducted in the context of various retention methods utilised by a given panel study, which may influence which covariates are associated with attrition for that particular study. For example, attrition analyses for longitudinal studies offering sizeable monetary rewards for responding may be less likely to find evidence of self-enhancement values being linked to attrition. This should therefore be kept in mind when considering the generalizability of our findings to other longitudinal studies.

Finally, it should be noted that, because of the breadth of the overall questionnaire, our measures of personality and attitudes were necessarily based on marker items and short-form scales. Although the personality measures used in this research have been extensively validated [2], [46], [47], the internal reliability for some of our short form scales were reasonably low. Indeed, the internal reliability of the three-item measure of felt belongingness was α = .53, whereas the reliability of other self-report measures generally ranged from α = .60 to α = .75. These relatively low levels of internal reliability may have attenuated our effect sizes, thus potentially underestimating the strength of personality and attitude factors in predicting attrition by a small amount.

### Concluding Comments

Previous research on panel attrition has tended to focus on demographic measures associated with attrition, with research on socio-psychological factors associated with attrition being scarce. Further, little (if any) research has been conducted with New Zealand longitudinal studies. In the present study we addressed these issues by documenting attrition in the New Zealand Attitudes and Values Study (NZAVS), a longitudinal national probability panel survey of New Zealand adults. Results indicated that Asian peoples and those with less education were most likely to have explicitly withdrawn. The permanent loss of these people from the sample may be cause for concern for both the NZAVS and other national panel studies. Retaining people from these demographic groups is important if panel studies are to remain representative. Moreover, many socio-psychological variables were associated with attrition, highlighting the importance of examining a range of factors associated with dropping out of longitudinal panel studies beyond mere demographics. These findings will help us to understand and manage attrition in the NZAVS as we move forward over the planned 20-year life-span of the study. We hope that these findings will also help further the understanding of which groups are difficult to retain in longitudinal studies more generally, and thus help inform the development of better strategies for maintaining panel retention and reducing selective attrition in longitudinal research.
